# Two-transcript gene expression classifiers in the diagnosis and prognosis of human diseases

**DOI:** 10.1186/1471-2164-10-583

**Published:** 2009-12-05

**Authors:** Lucas B Edelman, Giuseppe Toia, Donald Geman, Wei Zhang, Nathan D Price

**Affiliations:** 1 Institute for Genomic Biology, University of Illinois at Urbana-Champaign, Urbana, Illinois 61801, USA; 2 Department of Bioengineering, University of Illinois at Urbana-Champaign, Urbana, Illinois 61801, USA; 3 Department of Chemical and Biomolecular Engineering, University of Illinois at Urbana-Champaign, Urbana, Illinois 61801, USA; 4 Department of Applied Mathematics and Statistics & Institute for Computational Medicine, Johns Hopkins University, Baltimore, Maryland 21218, USA; 5 Department of Pathology, University of Texas MD Anderson Cancer Center, Houston, Texas 77030, USA; 6 Babraham Institute, Cambridge, CB22 3AT, UK

## Abstract

**Background:**

Identification of molecular classifiers from genome-wide gene expression analysis is an important practice for the investigation of biological systems in the post-genomic era - and one with great potential for near-term clinical impact. The 'Top-Scoring Pair' (TSP) classification method identifies pairs of genes whose relative expression correlates strongly with phenotype. In this study, we sought to assess the effectiveness of the TSP approach in the identification of diagnostic classifiers for a number of human diseases including bacterial and viral infection, cardiomyopathy, diabetes, Crohn's disease, and transformed ulcerative colitis. We examined transcriptional profiles from both solid tissues and blood-borne leukocytes.

**Results:**

The algorithm identified multiple predictive gene pairs for each phenotype, with cross-validation accuracy ranging from 70 to nearly 100 percent, and high sensitivity and specificity observed in most classification tasks. Performance compared favourably with that of pre-existing transcription-based classifiers, and in some cases was comparable to the accuracy of current clinical diagnostic procedures. Several diseases of solid tissues could be reliably diagnosed through classifiers based on the blood-borne leukocyte transcriptome. The TSP classifier thus represents a simple yet robust method to differentiate between diverse phenotypic states based on gene expression profiles.

**Conclusion:**

Two-transcript classifiers have the potential to reliably classify diverse human diseases, through analysis of both local diseased tissue and the immunological response assayed through blood-borne leukocytes. The experimental simplicity of this method results in measurements that can be easily translated to clinical practice.

## Background

The development of gene expression microarray technology has enabled genome-wide transcriptional profiling of human and other cells in diverse tissues and phenotypic contexts [1-5]. Among the most significant applications of global transcriptional profiling is the identification of molecular markers that provide accurate diagnosis, prognosis, and selection of treatment regimens for human disease [6-10]. Other important applications include elucidating biomolecular pathways that participate in pathogenic processes in order to identify potential targets for therapeutic intervention [11-13].

Recent investigations have generated quantitative classifiers that typically consider tens to hundreds of relevant genetic transcripts in the classification of different disease states or the analysis of pathogenic processes [14,15]. In particular, machine learning techniques such as support vector machines [16] and neural networks [17] have been applied to analyze transcriptional phenomena associated with disease progression, as well as with the prediction of patient prognoses and clinical response to therapy [18-21]. These methods are able to identify genes and gene networks associated with specific disease phenotypes, and thus provide a multivariate model for genetic perturbations involved in the generation and progression of disease.

The top-scoring pair (TSP) algorithm discriminates between binary phenotypic states using just two transcriptional measurements. First described by Geman and colleagues in 2004 [22], the TSP algorithm evaluates the relative expression of all possible pairs of genes in a microarray probe set, and selects those gene pairs for which the ordering of expression is most likely to reverse from one phenotype to the other. No numerical coefficients or parameters need be established through regression techniques. Exhibiting fewer degrees of freedom to be 'tuned' with experimental data, this method can consequently generate statistically significant classifiers with a comparatively smaller amount of microarray training data while generally avoiding problems of overfitting. Additionally, the algorithm is intrinsically invariant to monotonic data normalization, and can thus be more readily applied to different microarray platforms and probe sets. To discriminate between complex or closely-related phenotypes, 'k' different top-scoring pairs can be aggregated using a voting procedure to form a combinatoric 'k-TSP' classifier. Due to requirements for only a very small number of transcriptional measurements, the TSP and k-TSP methods embody a promising approach for identification of molecular markers that could be applied in the clinic.

TSP classifiers have been previously shown to accurately classify a number of human cancers, including tumors of the colon, prostate, and lung [23,24]; a transcriptional signature common to many cancers was also developed [25]. Notably, a robust two- gene classifier composed of OBSCN and PRUNE2 was found to differentiate between gastrointestinal stromal tumor (GIST) and leiomyosarcoma (LMS) with near-perfect accuracy in close to 100 patients tested [26]. Additionally, a two-transcript classifier was recently found to predict the response of acute myeloid leukemias to the small molecule therapeutic tipifarnib with high accuracy [27]. In the present study, we applied the TSP algorithm to construct accurate and statistically significant two-transcript classifiers for diverse diagnostic tasks, including the prediction of viral and bacterial infection, cardiomyopathies, metabolic disorders, and gastrointestinal ailments. The biological samples used in this study were obtained from both solid tissues and blood-borne immune cells. We observed that the TSP method not only compares favourably to pre-existing transcription-based statistical classifiers, but in certain phenotypes performs with similar accuracy to clinical diagnostic methods.

## Results

### Implementation of the TSP and k-TSP Algorithm

We acquired publicly-available microarray gene expression data representing a diverse spectrum of human pathologies from the Gene Expression Omnibus [28]. These studies were conducted on human clinical specimens using commercially available microarray platforms, and were selected to represent a diversity of human diseases, tissue and organ systems, and experimental study procedures. Each classification task compared one disease phenotype against either a second disease condition or a healthy control (Table 1). Patient specimens were taken from tissue biopsies or isolated peripheral blood mononuclear cells (PBMC); mRNA was extracted and assessed with commercial microarray platforms. Additional information regarding these datasets is available (Additional File 1). We crafted a top-scoring pair algorithm, available upon request, using the commercially available Matlab programming environment (Mathworks Inc, Natick MA). The input to this integrated program is a microarray gene expression dataset representing a number of clinical specimens, with annotations of phenotypic class for each sample. It then assesses all possible gene pairs in the microarray platform, and ranks the gene pairs based upon how well relative expression correlates with phenotype. The program also assesses statistical significance by applying the algorithm to data for which the phenotype labels have been randomly permuted across all samples, thereby determining the likelihood of finding apparently accurate classifiers due to chance using a false-discovery rate calculation. Additionally, the program performs leave-one-out cross-validation (LOOCV) to estimate the performance of the algorithm on novel data.

**Table 1:** Diagnostic Classification Tasks

Classification Task	Tissue Source	Samples (Positive/Negative)	GEO ID	# Probes
GI Stromal Tumor vs Leiomyosarcoma	GI Biopsy	68 (37/31)	N/A	43,931

Crohn's Disease vs Healthy Controls	PBMC	101 (59/42)	GDS1615	22,283

Ischemic vs Idiopathic Cardiomyopathy	Cardiac Biopsy	194 (86/108)	GSE5406	22,283

Type I Diabetes vs Healthy Controls	PBMC	105 (81/24)	GSE9006	22,283

Type II Diabetes vs Healthy Controls	PBMC	35 (12/23)	GSE9006	22,645

Ulcerative Colitis W/WO Transformation	Colon Biopsy	54 (11/43)	GSE3629	54,681

Gram-Negative vs Gram-Positive Infection	PBMC	73 (29/44)	GSE6269	22,283

Gram-Negative vs Viral Infection	PBMC	62 (18/44)	GSE6269	22,283

HIV Infection vs Healthy Controls	PBMC	86 (74/12)	GDS1449	8793

Microarray gene expression datasets obtained from the Gene Expression Omnibus. Transcriptional analysis was performed on either local tissue biopsies or peripheral blood mononuclear cells using commercially available oligonucleotide probe arrays. For sensitivity and specificity analysis, gastrointestinal stromal tumor (GIST), ischemic cardiomyopathy, transformed ulcerative colitis, and viral infection were defined as 'positive' diagnoses.

### Expression Data Yields Non-Overlapping TSP and k-TSP Classifiers

**Figure 1:**
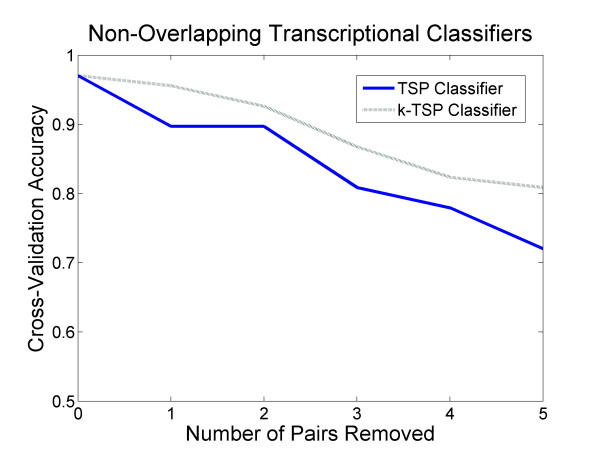
**Non-Overlapping TSP and k-TSP Classifiers for GIST and LMS Diagnosis**. Cross-validation accuracy of the k-TSP classifier as a function of top-scoring pairs being removed from microarray gene expression data of clinical GIST and LMS specimens. For k-TSP classification, k is held to a maximum of 11 pairs.

We sought to determine the degree to which TSP and k-TSP classifiers are sensitive to the transcriptional measurements available on microarray probe sets. In previous studies, highly accurate single- and multi-pair classifiers were generated in diverse cancer classification tasks. However, the extent to which other gene pairs are able to discriminate between phenotypic states, beyond these top-performing classifiers, has never been determined. We iteratively generated TSP and k-TSP classifiers on the same GIST/LMS microarray data originally used to derive the OBSCN/PRUNE2 classifier [26]. This dataset contains 68 clinical tissue specimens, and was assessed using a microarray with 43,931 oligonucleotide probes. Following each application of the search algorithm, we removed the top-scoring pair of genes from the dataset, and then repeated the algorithm to determine the accuracy of each classifier derived from the reduced dataset without the original best-scoring gene pairs. As seen in Figure 1, the TSP and k-TSP algorithms retain appreciable cross-validation accuracy even after the removal of multiple top-scoring gene pair classifiers, though there are reductions in performance. The presence of accurate non-overlapping transcriptional classifiers was also observed in other datasets examined using this process of iterative reduction of the probe set. The combinatoric k-TSP classifier achieved higher predictive accuracy upon removal of top-scoring pairs than the single-pair TSP classifier.

### Two-Transcript Classifier Accuracies in Diverse Diagnostic Tasks

The top-scoring pair algorithm generated classifiers that discriminate between diverse phenotypic states with various degrees of apparent accuracy (Table 2). We also examined the performance of the combinatoric k-TSP method on these datasets, and found that it outperformed the single-pair TSP method on some of the datasets using classifiers involving three to seven gene pairs (Table 3). Cross-validation accuracies, an estimation of algorithm performance on novel data, compared well with apparent top accuracy, with most LOOCV accuracies observed to be above 85% (Table 4). A lower classifier performance in cross-validation when the apparent accuracy is high does not necessarily imply that the functional accuracy of the algorithm for a particular phenotype separation is limited, but rather that the sample sizes obtained in these microarray studies may not be sufficient to determine the most accurate gene pairs for diagnosis. Of course, in instances where the observed cross validation accuracy is low, there is low confidence in the ability of the selected TSP to accurately classify future samples.

**Table 2:** Accuracy of Two-Transcript Classifiers on Diverse Phenotypes

Classification Task	Accuracy (Sens./Spec.)	Classifier Gene Pair and Annotated Functions	False Discovery
GIST/LMS	100% (100.0/100.0)	PRUNE2 (Regulation of Apoptosis) OBSCN (Muscle Differentiation & Signaling)	< 10 E-5

Crohn's Disease	96.04% (96.6/95.2)	TBX21 (Immune Modulation) APOLD1 (Angiogenesis; Lipid Metabolism)	< 10 E-5

Cardiomyopathy	74.23% (58.1/87.0)	PDE8B (Phosphodiesterase; cAMP Metabolism) ZNF263 (Zinc-Finger Transcription Factor)	< 0.002

Type I Diabetes	91.43% (96.3/75.0)	CD1D (Antigen Processing and Presentation) PSD (ARF/RAS Signal Transduction)	< 0.002

Type II Diabetes	100% (100.0/100.0)	UNC5A (Regulation of Apoptosis) ATG16L2 (Protein Transport; Autophagy)	< 0.005

UC Transformation	96.3% (81.8/100.0)	PAK2 (Kinase Signaling; Cell Cycle Regulation) FLT3LG (Immune Activation)	0.05910

Gram-Negative/Viral	100% (100.0/100.0)	CD40 (Immune Response; B Cell Proliferation) SETD6 (Histone Methyltransferase Activity)	< 10 E-4

HIV Infection	100% (100.0/100.0)	GAD1 (Glutamic Acid Metabolism) RHD (Erythrocyte Function)	< 10 E-4

Top apparent accuracy, sensitivity, and specificity, and false-discovery rate for each dataset using a two-gene TSP classifier. False discovery rate was based on the distribution of classifier accuracies following ten-fold random permutation of class labels.

**Table 3:** Accuracy of k-TSP Classifiers

Classification Task	Apparent Accuracy	Cross-Validation	Optimal K
GIST/LMS	100.00%	97.06%	3

Crohn's Disease	98.00%	91.10%	7

Cardiomyopathy	85.10%	65.00%	7

Type I Diabetes	90.50%	82.70%	3

Type II Diabetes	100.00%	82.70%	3

UC Transformation	98.20%	88.90%	3

Gram-Negative/Viral	100.00%	100.00%	3

HIV Infection	98.80%	94.20%	3

Top apparent accuracy, leave-one-out cross-validation performance, and optimal 'k' value for each dataset achieved by the combinatoric k-TSP algorithm.

**Table 4:** Cross-Validation Accuracy of Two-Transcript Classifiers

Classification Task	CV Accuracy	CV Sensitivity	CV Specificity
GIST/LMS	97.06%	93.55%	100.00%

Crohn's Disease	87.13%	88.14%	85.71%

Cardiomyopathy	74.23%	58.14%	87.04%

Type I Diabetes	91.43%	96.30%	75.00%

Type II Diabetes	94.29%	91.67%	95.56%

UC Transformation	83.33%	36.36%	95.35%

Gram-Negative/Viral	96.77%	88.89%	100.00%

HIV Infection	88.37%	90.54%	75.00%

Leave-one-out cross-validation accuracy, sensitivity, and specificity for each dataset using a two-gene TSP classifier.

Sensitivity and specificity were found to vary with the dataset. Two cases exhibit markedly low sensitivity - cardiomyopathy and transformed colitis. This was likely due to the comparatively smaller number of "positive" than "negative" tissue samples present in these microarray datasets, which serves as an implicit 'weight' for the algorithm to selectively choose classifiers exhibiting correct 'negative' diagnoses over correct 'positive' diagnoses. Variability in cross-validation accuracy was observed as a function of the disease being examined, with limited correlation observed between sample size and classifier accuracy. Extremely low false-discovery rates were witnessed for all datasets as derived from comparing the distributions of classifier accuracies in unmodified and randomly permuted data, indicating high statistical significance of each classifier. Every classifier except the prediction of ulcerative colitis transformation had an estimated false discovery rate of well below 0.01 from 10 independent permutations; the lower performance in this dataset was likely due to the smaller number of experimental samples included therein.

**Figure 2:**
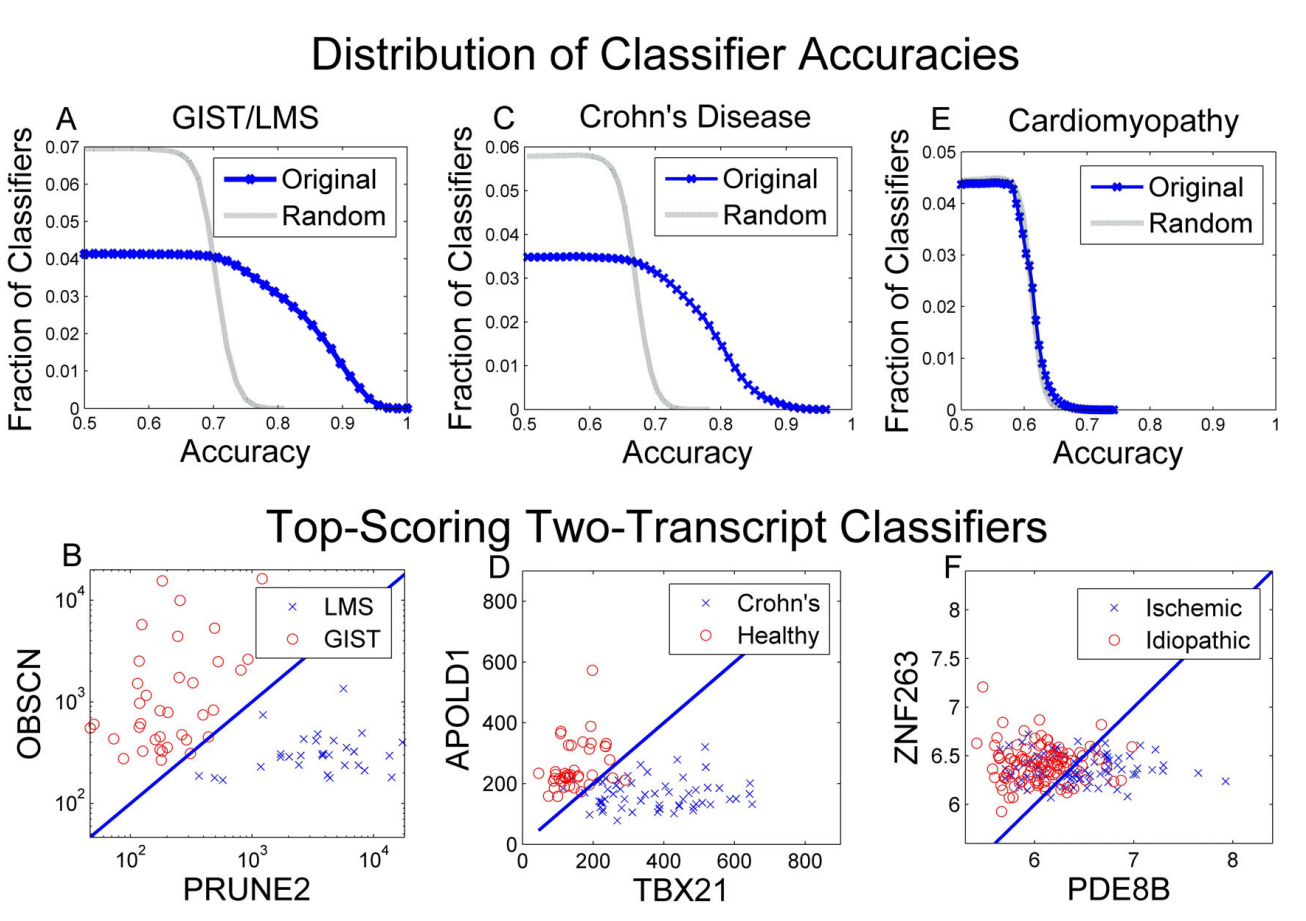
**Top-Scoring Classifiers and Distributions of Classifier Accuracies**. **A: **The distribution of all possible gene pair classifiers according to accuracy in the diagnosis of GIST and LMS, for both original data and randomly permuted data with randomized class labels. Vertical axis represents the fraction of pairs achieving the indicated accuracy. **B: **Plot comparing the expression level of the two genes from the top-scoring classifier as measured through a microarray platform, with a line of slope one and intercept of zero separating the two phenotypes according to the more highly expressed transcript. (Figure adapted from data originally published in [26].) **C and D: **Classifier accuracy distribution and top-scoring classifier microarray gene expression values for the diagnosis Crohns's Disease from circulating leukocytes. **E and F: **Accuracy distribution and top-scoring classifier in the diagnosis of ischemic and idiopathic cardiomyopathy.

Selected classification tasks are shown in Figure 2, including the distribution of gene-pair accuracy, and a graphical representation of top-scoring classifiers. As would be expected, the vast majority of gene pairs have low predictive accuracy in the given classification tasks, with only a small fraction exhibiting strong correlation with phenotype. Importantly, the random permutation of class labels sharply reduces the apparent accuracy of the classification algorithm for most datasets, indicating that the classifiers derived on original, unmodified data are statistically significant, corresponding to true molecular separation of the two phenotypes rather than being a product of chance. These results compare favourably with classifiers reported for these datasets using other statistical classification methods.

## Discussion

We have shown that simple two-transcript gene expression classifiers can accurately classify a wide spectrum of human diseases. This algorithm is invariant to data normalization and generates robust, statistically significant biological classifiers even in the context of low sample sizes. Our results reveal that many pathological processes, even those not traditionally considered genetic in nature such as infections and inflammatory disorders, can be diagnosed through just two transcriptional measurements. Whereas previous work has shown the diagnostic value of gene expression perturbations, this study demonstrates that as few as two transcriptional measurements can reliably detect diverse human diseases.

Transcriptional networks themselves can thus be seen to encode aspects of pathological phenotypes, with strong correlation observed between gene expression status and disease state. These transcriptional signatures were sufficiently robust to be detected even in tissue samples of possibly heterogeneous cell populations. The accuracies observed in these simple diagnostic modalities were comparable to pre-existing transcription-based classifiers that rely on more complex, multivariate measurements. For example, a 12-gene classifier generated against the same Crohn's disease dataset using a weighted-voting scheme exhibited a cross-validation accuracy of 94%, compared with equivalent TSP cross-validation performance of 87% [29]. Additionally, a 35-gene k-Nearest-Neighbor classifier trained on the same viral and bacterial infection dataset achieved a cross-validation accuracy of 91%, compared with 96% for the TSP approach [30].

The TSP method compared favourably to the estimated accuracy of standard clinical methods for the differentiation of viral and bacterial infection, as well as cardiomyopathy classification- conditions that present ongoing diagnostic challenges in the clinic. For example, a recently developed clinical prediction rule to discriminate between bacterial and viral pneumonia in children achieved positive predictive value of under 80%, in contrast to a TSP classifier cross-validation accuracy of 96.7% [31]. Additionally, a recent study of over 1200 patients presenting with diverse cardiomyopathies found that no pathologic etiology could be definitively elucidated in over 50% of clinical cases, in comparison with a cross-validation accuracy of over 70% achieved by the corresponding TSP classifier [32]. These results do not imply that the TSP method provides intrinsically superior diagnostic discrimination to 'gold standard' clinical measures - the TSP classifiers themselves are constrained by the fidelity of clinical methods used to diagnose patient samples contained within their respective training datasets. However, these results do indicate that properly trained TSP classifiers may exhibit higher accuracy in medical contexts where high-fidelity diagnoses are difficult or impractical to regularly obtain using other methods.

Interestingly, the ability of the classifier to obtain an accurate diagnosis was significantly lower in the comparison of ischemic and idiopathic cardiomyopathies than in any other dataset we examined. This is likely due to the broad cellular and metabolic heterogeneity observed in these two closely related conditions. Both clinical and molecular differentiation of ischemic and idiopathic cardiomyopathies remains a significant challenge [33]. Ischemic cardiomyopathy is diagnosed when oxygen delivery to the myocardium is inhibited, most often due to coronary artery disease. However, the presence of this condition is not diagnosed with great precision in the clinic, and idiopathic cardiomyopathy is diagnosed when no etiological factor for cardiovascular dysfunction can be explicitly isolated [32]. The failure of the algorithm to accurately discriminate between these two conditions may indicate that they represent overlapping genetic and physiological states, or that their respective diagnoses are not made with high fidelity in clinic, or a combination of both factors. This molecular heterogeneity has recently been confirmed using alternative gene expression analysis methods [34]. It is possible that other factors, such as consistency of tissue collection and processing, may negatively impact the quality of microarray data and thus the apparent performance of the algorithm. It is also possible that the two-transcript classifier scheme does not capture pathological information encoded by other molecular media - for example, protein or metabolite levels - that may more accurately predict pathological state. However, it is clear that a chief factor constraining the performance of the TSP cardiomyopathy classifier is the low fidelity of diagnostic decisions upon which it was trained. In the phenotypes studied where higher clinical diagnostic efficacy is achieved, the TSP classifier exhibits likewise higher accuracy.

We observed that the genes present in highly accurate two-transcript classifiers were often associated with disease processes in previous literature reports. For example, PRUNE2 has been shown to inhibit certain forms of oncogenic transformation, which may correspond to its differential regulation in GIST and LMS as observed through the TSP method [35]. The TSP prediction rule to diagnose Type I Diabetes is based on the relative expression of the genes CD1D and PSD. CD1D is a transmembrane protein involved in the presentation of lipid antigens to T cells and known to contribute to the generation of diabetes, and PSD belongs to a family of intracellular signal transduction proteins known to increase insulin sensitivity [36-39]. The change in expression of these two genes within the classifier thus recapitulates the underlying molecular etiology of the disease. While not all genes in the classifiers found through this study were known *a priori *to be involved in pathological processes, the strong association held by many such transcripts with their cognate phenotypes demonstrates the biomolecular relevance of these classifiers.

Intriguingly, in this study it was found that analysis of transcription in circulating mononuclear cells provides a robust diagnostic platform for both the detection of invading cellular or viral pathogens, and the diagnosis of somatic medical conditions such as diabetes and Crohn's Disease. Of particular interest are the simplicity, robustness and accuracy of two-transcript classifiers using a data source that provides an easily accessed transcriptomic 'readout' from pathologies of disparate tissues. Recent studies have examined the utility of serum-borne mRNA in the prediction of diseases, with varying fidelity [40,41]. These methods are constrained by the finite stability of RNA transcripts in the circulation. In contrast, the metazoan immune system exhibits an intrinsic and long-lasting 'memory' of cellular and other interactions that can persist in circulating cells for long periods. The interrogation of leukocyte gene expression would provide an easily deployed method for clinical diagnosis which, as indicated by these results, might present an informative discriminative measure in the diagnosis of diverse human diseases.

To implement the two-transcript classifiers, transcriptional measurements can be readily obtained in the clinic through routine PCR procedures [42]. The success of previous two-transcript diagnostics shows that, despite being formulated using microarray platforms, these intrinsically simple classifiers can be implemented efficiently through pre-existing gene expression methodologies. These classifiers therefore embody a promising platform for diverse diagnostic and prognostic tasks. These results also raise the exciting possibility that widespread human diseases could be reliably diagnosed through the acquisition of standard blood samples, a major objective of personalized medicine [43,44]. Sufficient information about the state of somatic tissues and organs may be encoded by the circulating leukocyte transcriptome to create a 'battery' of gene expression measurements that could simultaneously diagnose a large number of medical conditions. Further research is warranted to examine the degree to which different human pathologies could be inferred using simple transcriptional measurements from circulating cells.

## Conclusion

We have shown that the top-scoring pair algorithm is able to generate statistically significant and accurate gene expression classifiers from microarray data. These methods are insensitive to data normalization, and perform consistently when applied to novel experimental data. Furthermore, the method is able to detect diverse human diseases, even those not considered genetic in nature or cause. Ultimately, two-transcript classifiers obtained from microarray gene expression data present a robust analytical tool for clinical diagnostics.

## Methods

### Top-Scoring Pair Algorithm

The input to the top-scoring pair (TSP) algorithm is a gene expression matrix from a microarray probe set corresponding to semi-quantitative transcriptional measurement, from multiple unique tissue samples. The algorithm first replaces the gene expression value within each sample by its corresponding *rank *relative to all the gene expression values within the sample. This rank-based processing renders the algorithm invariant to monotonic data normalization. Importantly, this algorithm treats each probe within a microarray platform individually - such that, even when multiple probes are spotted independently for the same gene on a microarray, both probes are treated as independent, unrelated measurements.

The algorithm then assesses all possible pairs of genes *A *and *B *whereby their relative expression predicts phenotypic class (either class *1 *or *2*), employing a simple classification rule for any sample:

IF *Rank (gene A) > Rank (gene B)*, THEN Class = 1; ELSE Class = 2

For each gene pair, the number of accurate class predictions is counted and each gene pair is then ranked according to the cumulative predictive accuracy across all samples. The most accurate transcript pairs are returned as top-scoring classifiers. The mean *difference *in rank between two genes is calculated in the event of ties between equivalently accurate classifiers as described previously [22]. Sensitivity (defined as the proportion of true 'positive' diagnoses that are accurately detected by the classifier) and specificity (the proportion of true 'negative' diagnoses that are accurately detected) are also recorded for each top-scoring classifier.

To address the significant data-storage matters arising from assessing so many prospective classifier pairs, the algorithm employs a dynamic data analysis feature whereby only pairs that might possibly represent 'top-scoring' pairs are recorded for further analysis. Additionally, the algorithm employs this parsimonious library of highly accurate pairs to reduce the computational time required for the k-TSP algorithm and leave-one-out cross-validation analysis. With these optimizations, the algorithm is able to fully analyze even large microarray datasets within one day on a standard desktop computer, including cross-validation analysis and False Discovery Rate prediction.

### Combinatoric k-TSP Algorithm

In an extension of the TSP algorithm, *k *individual TSP classifiers can be combined into a multi-pair 'k-TSP' classifier. In this approach, the TSP algorithm itself is performed, and all possible transcript pairs are ranked in order of their classification accuracy. The top *k *highest-ranked TSP pairs for a given classification task each represent one 'vote', with equal weight, for the class of each given sample; the final predicted class of each sample is the phenotype with the majority of votes. To avoid ties, k is restricted to odd numbers only; for this study the maximum value of k was held to 11. For each classification task, a leave-one-out cross-validation loop (described following) is employed to determine the optimal value of k.

### Analysis of Non-Overlapping TSP and k-TSP Classifiers

We employed TSP and k-TSP algorithms to determine the degree to which these methods can generate multiple unique gene expression-based classifiers. We first determined the optimal TSP and k-TSP classifiers against the previously mentioned GIST/LMS gene expression data. We then removed the top-scoring individual gene pair from the dataset, and repeated the algorithm on this reduced gene expression data. We iteratively performed this gene-pair excision, and recorded TSP and k-TSP classifier accuracies at each step. The value of k was held to a maximum of 11, and was determined in each iteration by an internal loop of leave-one-out cross-validation that established the optimal value of k for each classification task.

### Leave-One-Out Cross-Validation

To estimate algorithm performance on novel samples, we performed leave-one-out cross-validation (LOOCV), in which the top-scoring pair as determined by *N-1 *samples is used to predict the left-out sample class. This cross-validation is performed iteratively for each of *N *samples, with the number of correct predictions out of *N *then averaged to determine LOOCV accuracy. Cross-validation sensitivity and specificity were also determined.

### Calculation of False-Discovery Rate

To estimate the statistical power of each classifier, we applied the algorithm to each dataset following random permutations of phenotypic class labels across all samples. We then compared the distribution of gene pair classification accuracy between actual and randomized data, and calculated the false discovery rate (FDR), corresponding to the likelihood of finding an apparently accurate classifier due to chance. Examples of these original and randomized distributions are shown in figure 2A, 2C, and 2E. The FDR estimate for any given accuracy cutoff was computed as FDR = FP/TP, where FP represents the false positive estimate at the selected accuracy cutoff, and TP represents the total positives (pairs above the selected accuracy cutoff observed in the dataset under consideration). The FP estimate was calculated by evaluating the accuracy of all gene pairs from 10 random permutations of the class labels for each phenotype comparison dataset considered herein. The FP estimate was computed as the average number of pairs above the cutoff accuracy observed in the 10 permutations. With random phenotype label permutations, we assume that all pairs observed above a given accuracy in these datasets should be considered as false positives. Because all pairs are considered for each permutation, the total number of pair accuracies considered for the null distributions is high (typical number per permutation for e.g. a human Agilent microarray would be around a billion). The FDR method accounts for the multiple hypothesis testing inherent in the TSP algorithm. In several cases of this study, no classifier in the accuracy distribution of randomized data achieved the top accuracy of those from the original data and thus these TSPs technically exhibited a calculated FDR of zero. In these cases, the lowest non-zero FDR value was listed as an (often loose) upper-bound estimate for the likely true FDR.

## Authors' contributions

LBE was responsible for the design and execution of the study, implemented the TSP algorithm, collected and processed data, and assembled the manuscript; GT assisted with the collection of microarray data and data analysis; WZ and NDP conceived of the study. WZ, DG, and NDP provided guidance on the study and edited the manuscript. All contributors read and approved the final manuscript.

## Supplementary Material

Additional file 1 **Appendix 1**. Datasets Click here for file
