# Supplementary material for: Two-transcript gene expression classifiers in the diagnosis and prognosis of human diseases
Source: BMC Genomics. 2009 Dec 5;10:583. doi: 10.1186/1471-2164-10-583 (PMC2797819; doi:10.1186/1471-2164-10-583)
Supplement: Additional file 1 — Appendix 1. Datasets [file 1471-2164-10-583-S1.PDF]

Two-Transcript Gene Expression Classifiers in the Diagnosis and Prognosis of Human Diseases

Appendix 1: Microarray Gene Expression Datasets

We sought to evaluate the TSP algorithm against diverse diagnostic tasks in human medicine, and therefore acquired several microarray datasets representing diverse tissues and tissue types – including both solid tissues and circulating leukocytes. Additionally, we sought to evaluate the algorithm performance on both communicable diseases such as viral and bacterial infections, as well as somatic medical conditions such as Diabetes and Crohn's Disease. Lastly, we included datasets employing several different microarray platforms and probe sets, to ascertain the robustness of the computational method to the experimental platform employed for data acquisition.

The following is a description of the microarray datasets employed in this study. All microarray studies except for one – the GIST/LMS dataset, for which data was provided by the original authors of the study – were downloaded from the Gene Expression Omnibus; corresponding GEO dataset identifiers are shown in parentheses. GEO is a publicly accessible website containing several thousand microarray datasets for scientific use. This website and the studies included in the current manuscript can be obtained at: http://www.ncbi.nlm.nih.gov/geo/

Gastrointestinal Stromal Tumor (GIST) and Leiomyosarcoma (LMS)

This study consisted of 68 primary tumor biopsies snap-frozen following surgical excision from patients with GIST (considered the 'positive' phenotype, with n = 37 samples) or LMS (n = 31 samples), analyzed with an Agilent Technologies 44k human gene expression microarray. This original study derived a two-transcript classifier for this diagnostic task, which was found to compare favorably with standard histopathological measures for discriminating between the two related diseases.

Crohn's Disease (GDS1615)

This study examined samples of peripheral blood mononuclear cells (PBMCs) isolated from patients with Crohn's Disease, Ulcerative Colitis, and healthy status. We obtained gene expression profiles derived from patients diagnosed with Crohn's Disease (n = 59), as well as healthy patients (n = 42). Transcription in each sample was profiled with an Affymetrix Human Genome U133A Array with approximately 22,000 probes. This original study created a transcription-based diagnostic classifier able to distinguish between Crohn's Disease and Ulcerative Colitis using a panel of 12 genes.

Ischemic and Idiopathic Cardiomyopathies (GSE5406)

This study examined biopsies of left ventricular myocardium from patients with advanced stage ischemic or idiopathic cardiomyopathy during receipt of heart transplantation surgery, as well as several healthy controls from unused donor hearts. These tissues were analyzed with the Affymetrix Human Genome U133A Array containing approximately 22,000 probes. We obtained samples from ischemic cardiomyopathy patients (considered the 'positive' diagnosis, with n = 86 samples) and idiopathic cardiomyopathy patients (n = 108). This original study sought to determine genes differentially expressed within the two cardiomyopathy conditions in contrast to healthy patients.

Type I and Type II Diabetes Mellitus (GSE9006)

This study examined PBMCs isolated from healthy children, and children with Type I or Type II Diabetes. These samples were analyzed with Affymetrix Human Genome U133A and U133B arrays, each with approximately 22,000 probes. We compared samples from patients with Type I Diabetes (n = 81) with samples from healthy patients (n = 24), as well as patients with Type II Diabetes (n = 12) versus healthy control patients from the same microarray platform (n = 23). This study sought to profile gene expression changes observed in young patients diagnosed with different forms of Diabetes Mellitus to identify contributing transcriptional events in the pathogenesis of the disease, and identify prospective therapeutic targets.

Ulcerative Colitis (GSE3629)

This study examined samples obtained from surgically resected colon specimens from patients diagnosed with Ulcerative Colitis, with and without concomitant malignant transformation to colon cancer. Transcriptional profiling of these biopsies was performed with an Affymetrix Human Genome U133 Plus 2.0 Array containing approximately 54,000 unique probes. We obtained gene expression data for patients with transformed Ulcerative Colitis (n = 11) and non-cancerous Ulcerative Colitis (n = 43). This original study applied support vector machine (SVM) and k-Nearest Neighbor algorithms to elucidate a 40-gene signature able to differentiate between transformed and non-transformed ulcerative colitis.

Viral and Bacterial Infection (GSE6269)

This study examined PBMCs isolated from individuals with acute bacterial or viral infections, which were then analyzed with Affymetrix Human Genome U133A and U133 Plus 2.0 arrays, containing 22,000 and 54,000 probes respectively. We obtained expression profiles for gram-positive Staphylococcal infections (n = 29), gram-negative *e. coli* infections (n = 44), and influenza viral infections (n = 18). This original study performed a hierarchical clustering procedure to create a signature of 35 differentially expressed genes for the prediction of infection etiology.

Human Immunodeficiency Virus Infection (GDS1449)

This study examined PBMCs isolated from individuals with positive HIV diagnoses, and healthy HIV-negative patients. These samples were profiled using Affymetrix Human-Genome Focus Arrays with approximately 8800 probes. We obtained expression profiles for HIV seropositive individuals (n = 74) and HIV negative persons (n = 12). This study sought to reveal genes differentially regulated upon HIV infection and relevant to the pathogenesis of the disease.
